# Superior gluteal nerve injury following landmark‐guided corticosteroid injection for greater trochanteric pain: A case report

**DOI:** 10.1002/ccr3.3202

**Published:** 2020-08-09

**Authors:** Austin R. Thompson, Erik R. Ensrud

**Affiliations:** ^1^ Department of Orthopaedics and Rehabilitation Oregon Health & Science University Portland Oregon

**Keywords:** gluteus medius, greater trochanteric pain syndrome, nerve injection injury, superior gluteal nerve

## Abstract

This report describes an isolated superior gluteal nerve injection injury following a corticosteroid injection for greater trochanteric pain syndrome. Ultrasound‐guided injections may be beneficial to target multiple pain‐producing regions of the hip while avoiding nerves and tendons.

## Graphical abstract

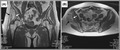

## 1 INTRODUCTION

Corticosteroid injections are an effective conservative treatment for greater trochanteric pain syndrome. This report describes the clinical presentation of a 64‐year‐old female patient with an isolated right superior gluteal nerve injury following a landmark‐guided corticosteroid injection of the trochanteric bursae.

Greater trochanteric pain syndrome (GTPS) presents as pain over the superior lateral region of the hip and tenderness to palpation over the greater trochanter. This term is considered preferable to greater trochanteric bursitis because there may not be signs of inflammation where the reported pain is located and pain may be related to numerus surrounding structures.^1^ A sonographic investigation of GTPS found that 80% of patients did not have evidence of bursitis on ultrasound.^2^ The patients that comprised the proportion without bursitis included 50% of patients with evidence of gluteal tendinosis and 29% of patients with iliotibial band thickening.^2^ In community‐dwelling adults ages 50‐79 years, GTPS has been estimated to have a prevalence as high as 23.5% in women and 8.5% in men.^3^

Conservative treatments for GTPS include activity modification or rest, nonsteroidal anti‐inflammatory medications, physical therapy, and corticosteroid injections.^4^ The anatomic structures targeted with corticosteroid injections are bursae, most commonly the greater trochanteric bursa or the subgluteus medius bursa. Injection techniques can vary with physician specialty, experience, and practice type.^5^ Both anatomic landmark‐ and ultrasound‐guided injection of these structures are commonly performed, with ultrasound‐guided injections becoming more popular. It has been suggested that anatomic landmark‐guided injections might not be as effective due to the proportion of injections that fail to reach the target area.^5^

Musculoskeletal injections are associated with potential complications. While complications are relatively rare, they may include allergic reactions, bleeding, infection, tendon rupture, or nerve injury.^6^ Injection‐associated nerve injuries may be a result of direct needle trauma or ischemia, and intrafascicular damage may result in more severe injury.^7^ We describe a case of superior gluteal nerve injury following anatomic landmark palpation‐guided corticosteroid injection for GTPS.

## 2 CASE PRESENTATION

A 64‐year‐old woman with no comorbidities and a body mass index of 24 kg/m^2^ presented to clinic for evaluation of her right hip and leg after developing weakness following multiple greater trochanteric bursa injections by an orthopedic surgeon at an outside institution. She reported that right‐sided hip pain started 6 months prior after prolonged sitting on a two week bus tour. Pain was provoked during periods of standing. She worked as a floor nurse, where pain severely limited her ability to complete her tasks. She was evaluated by the surgeon and diagnosed with right hip greater trochanteric bursitis. The patient provided the history that the orthopedic surgeon injected corticosteroid into the trochanteric bursae using anatomic landmarks. She reported that her symptoms temporarily improved with the injection but reoccurred. She stated that she continued to receive landmark‐guided corticosteroid injections from this provider every 4‐6 weeks. On the sixth landmark‐guided corticosteroid injection, she explained that the surgeon fanned out the injection from a single site in an effort to disperse the steroid over a larger area. The patient reported immediate pain and discomfort in the injection location. Within one week of the repeat injection, she experienced severe weakness of the right proximal leg. She was evaluated by a neurologist and had an electromyography (EMG) study performed. The EMG sampling included the right tibialis anterior but did not include the right gluteus medius; EMG results were unremarkable.

**Figure 1:**
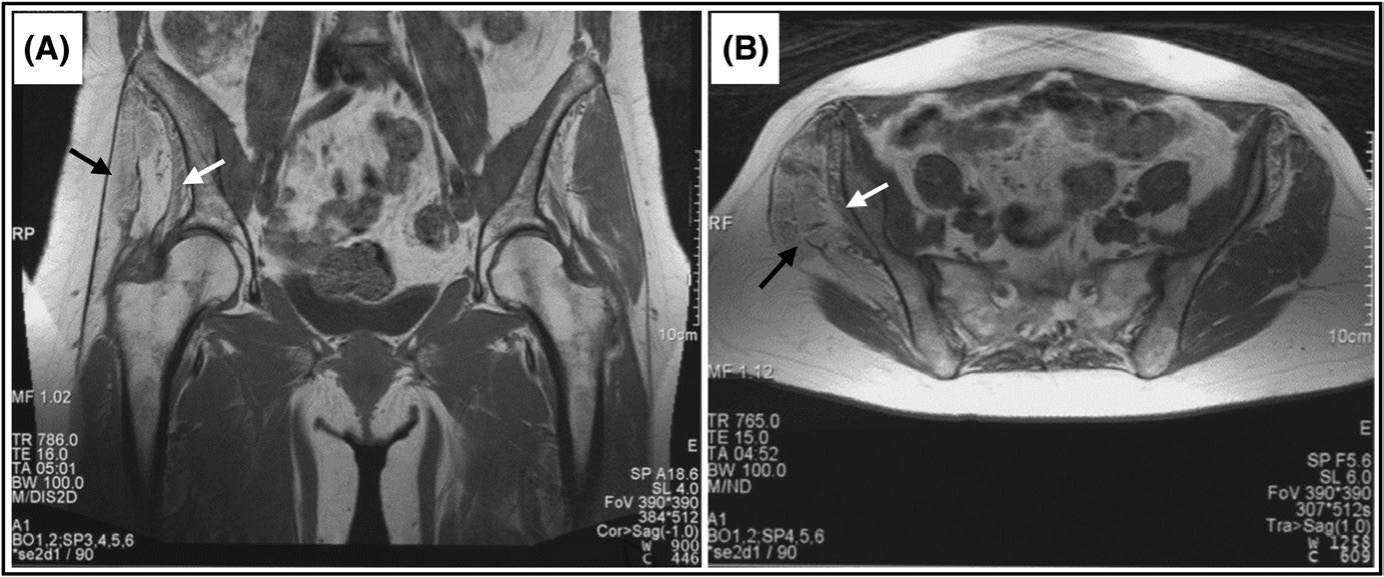
Magnetic resonance imaging of the pelvis. A, Coronal view and B, Axial view depicting diffuse fatty infiltration of the right gluteus medius and minimus. Gluteus medius, black arrows; gluteus minimus, white arrows

Upon physical examination at our clinic, the patient had normal muscle bulk in the thighs and calves bilaterally. Muscle strength was normal, with the exception of 3/5 Medical Research Council (MRC) score for right hip abduction, compared to 5/5 on the left. Ankle dorsiflexion was 5/5 bilaterally. Gait evaluation was notable for a markedly positive right Trendelenburg sign. Magnetic resonance imaging (MRI) 6 months after the final injection showed diffuse fatty infiltration and gross muscle atrophy in the right gluteus medius and minimus compared to the contralateral side (Figure 1). We conclude that the fanning injection method caused direct needle trauma of the right superior gluteal nerve, resulting in the immediate pain experienced and severe muscle atrophy in the superior gluteal nerve distribution.

Fifteen years after the injury, she remains very active by working with a physical trainer multiple times per week. She has developed compensatory mechanisms to account for the atrophied muscles. She still struggles some with balance and isolated hip abduction is still weaker on the right compared to left side.

## 3 DISCUSSION

The report describes a patient that developed symptoms and clinical findings consistent with a superior gluteal nerve injection injury following an anatomic landmark‐guided injection for GTPS. Clinical correlations of the physical examination, MRI, and EMG are used to diagnose nerve injuries. The gluteus medius is not normally examined in a standard lower extremity EMG, which is why the nerve injury may have been initially missed. However, upon MRI examination, extensive fatty infiltration of the right gluteus medius and gluteus minimus was noted. These findings are consistent with chronic muscle denervation.^8^ The MRI findings and positive right Trendelenburg sign with associated hip abductor weakness support the diagnosis.

Isolated superior gluteal nerve injuries have been rarely described,^9^, ^10^, ^11^ with a couple reports describing injection‐related injuries.^12^, ^13^ Lower extremity nerve injection injuries are most commonly seen in the sciatic nerve; however, similar factors associated with nerve injection injuries can be seen with the gluteal nerve.^7^ Injection of corticosteroids or other drugs may induce scar formation, which may lead to fibrosis and constrict nerves as well as surrounding tissues, limiting blood flow. Neural ischemia may occur as a result. It is believed that this type of nerve injection injury tends to have a delayed onset.^7^ In contrast, direct intraneural needle trauma is associated with an immediate onset of symptoms, which was observed in the patient presented in this case report.

If there is clinical concern of multiple pain‐producing regions surrounding the hip, it may be beneficial to use ultrasound guidance during injections to avoid damaging anatomical structures and to confirm injection locations. Ultrasound‐guided injections can provide more precise injections into the greater trochanteric bursa as well as the subgluteus medius bursa.^14^ When injecting other locations thought to be the source of the pain, ultrasound‐guided injections may be limited in visualizing the needle when injecting deep anatomical regions.^14^ While ultrasound‐guided injections might not be cost‐effective for routine injections of trochanteric bursae, ultrasound‐guided injection may be useful in certain patient populations, that is, in extremely obese populations or in cases where prior anatomic landmark‐guided injections failed.^15^ Careful consideration should be used to accurately and effectively administer corticosteroid injections to the pain‐producing regions contributing to GTPS.

This report is limited by the fact that many of the initial diagnostic and treatment details are patient reported since the initial evaluation and injections were performed elsewhere and not available for our review. However, this report is applicable to clinical practice as some patients present to clinic without prior medical records and the evaluation and management is dependent on their reporting. Additionally, this information would not change the diagnosis of the superior gluteal nerve injection injury nor our conclusion that ultrasound‐guided injection may have prevented nerve injury by allowing the visualization of the needle during the injection administration.

Isolated superior gluteal nerve injection injuries may occur with injections for greater trochanteric pain syndrome. Ultrasound‐guided injections may reduce superior gluteal nerve injection injuries by confirming injection locations and visualizing the needle throughout injection administration. However, when these injection injuries do occur, careful clinical correlation of physical, electrodiagnostic, and imaging findings should be used when evaluating patients that report lateral hip pain with abductor weakness following musculoskeletal injections.

## CONFLICT OF INTEREST

None declared.

## AUTHOR CONTRIBUTIONS

AT: conducted the literature search on the topic and drafted the initial version of the manuscript. EE: collected clinical information and provided critical revision of the manuscript for intellectual content.
